# Statin, Calcium Channel Blocker and Beta Blocker Therapy May Decrease the Incidence of Tuberculosis Infection in Elderly Taiwanese Patients with Type 2 Diabetes

**DOI:** 10.3390/ijms160511369

**Published:** 2015-05-18

**Authors:** Mei-Yueh Lee, Kun-Der Lin, Wei-Hao Hsu, Hsiu-Ling Chang, Yi-Hsin Yang, Pi-Jung Hsiao, Shyi-Jang Shin

**Affiliations:** 1 Department of Internal Medicine, Kaohsiung Municipal Hsiao-Kang Hospital, Kaohsiung 812, Taiwan; E-Mails: lovellelee@hotmail.com (M.-Y.L.); my345677@yahoo.com.tw (W.-H.H.); 2 Division of Endocrinology and Metabolism, Department of Internal Medicine, Kaohsiung Medical University Hospital, Kaohsiung 807, Taiwan; E-Mail: 890073@kmuh.org.tw; 3 Department of Nursing, Kaohsiung Municipal Hsiao-Kang Hospital, Kaohsiung 812, Taiwan; E-Mail: 0880170@kmhk.org.tw; 4 Statistical Analysis Laboratory, Department of Clinical Research, Kaohsiung Medical University Hospital, Kaohsiung 807, Taiwan; E-Mail: yihsya@kmu.edu.tw; 5 Center for Lipid and Glycomedicine Research, Kaohsiung Medical University, Kaohsiung 807, Taiwan

**Keywords:** statin, beta blocker, calcium channel blocker, tuberculosis, elderly, diabetes

## Abstract

Background: It is well known that diabetes mellitus impairs immunity and therefore is an independent risk factor for tuberculosis. However, the influence of associated metabolic factors, such as hypertension, dyslipidemia and gout has yet to be confirmed. This study aimed to investigate whether the strong association between tuberculosis and diabetes mellitus is independent from the influence of hypertension and dyslipidemia, and its treatment in elderly Taiwanese patients. Methods: A total of 27,958 patients aged more than 65 years were identified from the National Health Insurance Research Database (NIHRD) in 1997 and were followed from 1998 to 2009. The demographic characteristics between the patients with and without diabetes were analyzed using the χ2 test. A total of 13,981 patients with type 2 diabetes were included in this study. Cox proportional hazard regression models were used to determine the independent effects of diabetes on the risk of tuberculosis. Results: After adjusting for age, sex, other co-morbidities and medications, calcium channel blocker, beta blocker and statin users had a lower independent association, with risk ratios of 0.76 (95% CI, 0.58–0.98), 0.72 (95% CI, 0.58–0.91) and 0.76 (95% CI, 0.60–0.97), respectively. Conclusion: Calcium channel blocker, beta blocker and statin therapy may decrease the incidence of tuberculosis infection in elderly Taiwanese patients with type 2 diabetes.

## 1. Introduction

The incidence of diabetes is increasing worldwide, and the global prevalence is expected to rise by 55% from the current 382 million to 592 million people by 2035 [1]. Although the prevalence is similar in both high- and low-income countries, over 80% of diabetes-related deaths occur in low- and middle-income countries [1]. More than nine million people develop tuberculosis (TB) every year and over 1.5 million die from TB every year, and the majority of deaths occur in the developing world [2]. In 2012, more than 20 million people worldwide were infected with MTB (mycobacterium tuberculosis), including 8.6 million new cases and 1.3 million deaths [3]. One in three people worldwide is infected with latent TB, and people infected with latent TB have a lifelong risk of developing active TB [2]. Aging is a complex process characterized by a gradual decline in organ functional reserves, which eventually reduces the ability to maintain homeostasis [4]. The autoimmune theory proposes that the immune system eventually fails to distinguish self from non-self antigens with aging [5]. People with a weak immune system, for example as a result of chronic diseases such as diabetes, are at a two to three-fold higher risk of progressing from latent to active TB compared to those without diabetes [6]. About 10% of TB cases globally are linked to diabetes, however a large proportion of people who have both diabetes and TB are not diagnosed or are diagnosed too late [7]. Early detection can help improve care and control of both diseases, and everyone with TB should be screened for diabetes, particularly in areas with a high prevalence of TB. People with diabetes who are diagnosed with TB have been reported to have a higher risk of death during TB treatment, and of TB relapse after treatment [8]. Treatments recommended by the World Health Organization (WHO) should be rigorously implemented for people with both TB and diabetes. Diabetes is complicated by the presence of infectious diseases including TB, and so it is important that proper care for diabetes is provided to those suffering from both TB and diabetes. Diabetes is a multifactorial complex disease with a host of associated metabolic and hormonal derangements characterized by hypertension, dyslipidemia and hyperglycemia, making treatment a challenge [9]. At present, chemotherapy alone cannot overcome the challenges for the treatment of tuberculosis, and therefore new treatment methods are needed. This study enrolled a large nationally representative cohort selected from the National Health Insurance (NHI) program of Taiwan with the aim of exploring the possible effects of hypertension, dyslipidemia, diabetes mellitus and their treatment on developing tuberculosis in elderly Taiwanese patients.

## 2. Results

**Figure 1:**
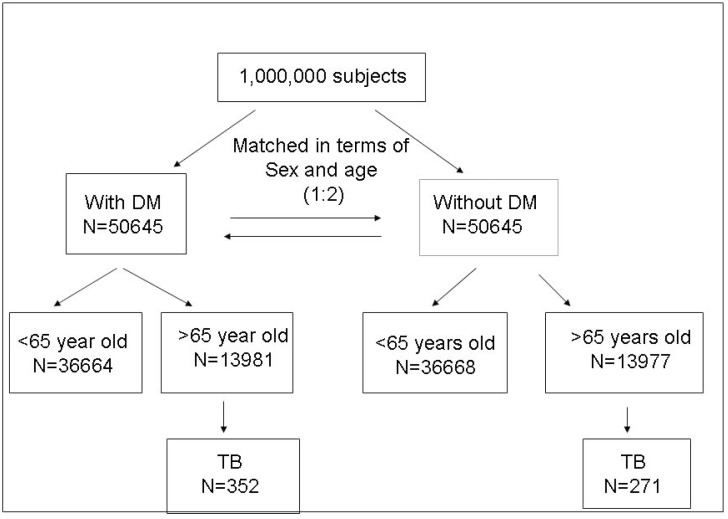
Overall framework of the research design and sampling strategy. DM = diabetes mellitus; TB = tuberculosis.

Of the one million subjects in the database, 50,645 were identified as having diabetes. Patients in the study group without diabetes were matched with the study groups in terms of age, sex, and the year and month of index visit, and also included 50,645 cases. Overall, 27,958 patients were older than 65 years (13,981 and 13,977 patients in the diabetes and no diabetes groups, respectively), and were followed up from 1998 to 2009 (Figure 1). The distributions of sex, age, income category, residence, prevalence of different co-morbidities, anti-diabetic, anti-hypertensive and anti-hyperlipidemia agents used in the patients with and without diabetes mellitus are shown in Table 1. Among the 13,981 patients with diabetes aged over 65 years, 6438 (46.0%) were male and 7543 (54.0%) were female. Most (58.4%) were employed with a monthly income of <20,000 New Taiwan Dollar (NTD). There were higher prevalence rates of gout (4.3% *vs.* 2.4%), hypertension (27.8% *vs.* 17.1%), hyperlipidemia (8.3% *vs.* 5.0%), asthma (5.3% *vs.* 3.2%), chronic obstructive pulmonary disease (COPD) (10.8% *vs.* 8.0%), end stage renal disease (1.5% *vs.* 0.9%), heart failure (4.6% *vs.* 2.0%), other cardiovascular diseases (9.4% *vs.* 5.6%) and tuberculosis (2.6% *vs.* 2.0%) in the patients with diabetes compared to those without diabetes. There were also higher prevalence rates of the use of angiotensin-converting-enzyme inhibitors (ACEI) (39.1% *vs.* 18.7%), angiotensin II receptor blockers (ARB) (49.8% *vs.* 25.3%), beta blockers (50.0% *vs.* 35.2%), calcium channel blockers (72.1% *vs.* 51.2%), diuretics (49.0% *vs.* 29.7%), hydralazine plus nitrate (0.4% *vs.* 0.2%), isosorbide (16.6% *vs.* 9.0%), other anti-hypertensives (38.5% *vs.* 25.7%), statins (33.7% *vs.* 12.3%) and other anti-hyperlipidemia agents (15.9% *vs.* 4.1%) in the patients with diabetes compared to those without diabetes.

**Table 1:** Demographic characteristics between those with and without diabetes aged >65 years.

Variables	Total	DM	Without DM	*p* Value
	*N*	*N* (%)	*N* (%)	
**Sex**				
Female	15,083	7543 (54.0%)	7540 (53.9%)	
Male	12,875	6438 (46.0%)	6437 (46.1%)	0.9919
**Income category**				
unemployed	10,374	5220 (37.3%)	5154 (36.9%)	
<20,000 NTD	16,592	8168 (58.4%)	8424 (60.3%)	
>20,000 NTD	992	593 (4.2%)	399 (2.9%)	<0.0001
**Residence**				
Metropolitan	6054	3028 (21.7%)	3026 (21.6%)	
Northern cities	1893	975 (7.0%)	918 (6.6%)	
Southern cities	1067	518 (3.7%)	549 (3.9%)	
Northern counties	12,401	6180 (44.2%)	6221 (44.5%)	
Southern counties	6421	3232 (23.1%)	3189 (22.8%)	0.1270
**Co morbidities**				
Gout	928	597 (4.3%)	331 (2.4%)	<0.0001
Hypertension	6285	3893 (27.8%)	2392 (17.1%)	<0.0001
Hyperlipidemia	1858	1162 (8.3%)	696 (5.0%)	<0.0001
Asthma	1190	736 (5.3%)	454 (3.2%)	<0.0001
COPD	2629	1507 (10.8%)	1122 (8.0%)	<0.0001
AIDS	1	1 (0.01%)	0 (0.00%)	0.3174
Connective tissue disease	399	217 (1.6%)	182 (1.3%)	0.0781
End stage renal disease	329	204 (1.5%)	125 (0.9%)	<0.0001
Heart failure	931	647 (4.6%)	284 (2.0%)	<0.0001
Other cardiovascular disease	2093	1309 (9.4%)	784 (5.6%)	<0.0001
Tuberculosis	623	352 (2.6%)	271 (2.0%)	0.0006
**Medications**				
Sulfonylureas	10,212	10,212 (73.0%)	-	<0.0001
Metformin	8508	8508 (60.8%)	-	<0.0001
Acarbose	2502	2502 (17.9%)	-	<0.0001
Thiazolidinediones	1305	1305 (9.3%)	-	<0.0001
Meglitinides	2508	2508 (17.9%)	-	<0.0001
Insulin	1531	1531 (10.9%)	-	<0.0001
ACEI	8083	5472 (39.1%)	2611 (18.7%)	<0.0001
ARB	10,578	6969 (49.8%)	3609 (25.3%)	<0.0001
Beta blockers	11,916	6991 (50.0%)	4925 (35.2%)	<0.0001
Calcium channel lockers	17,240	10,078 (72.1%)	7162 (51.2%)	<0.0001
Diuretics	11,007	6857 (49.0%)	4150 (29.7%)	<0.0001
Hydralazine + nitrate	76	49 (0.4%)	27 (0.2%)	0.0115
All nitrates	36	23 (0.2%)	13 (0.1%)	0.0955
Isosorbide	3586	2326 (16.6%)	1260 (9.0%)	<0.0001
Other anti-hypertensives	8977	5379 (38.5%)	3598 (25.7%)	<0.0001
Statins	6441	4715 (33.7%)	1726 (12.3%)	<0.0001
Other anti-hyperlipidemia agents	2796	2222 (15.9%)	574 (4.1%)	<0.0001

ACEI = angiotensin-converting-enzyme inhibitor; AIDS = acquired immune deficiency syndrome; ARB = angiotensin II receptor blocker; COPD = Chronic obstructive pulmonary disease; DM = diabetes mellitus.

**Figure 2:**
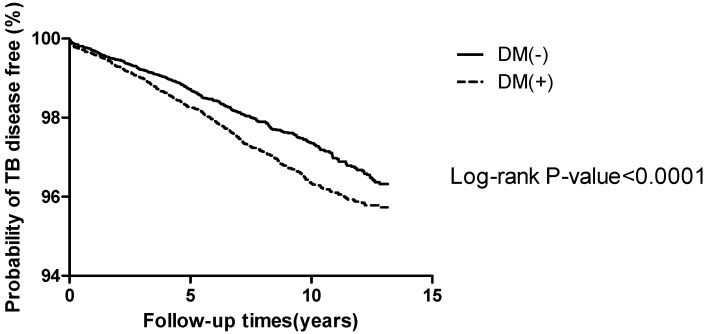
Kaplan–Meier curve for the patients with and without diabetes for the time to tuberculosis infection. DM = diabetes mellitus; TB = tuberculosis.

During the follow-up period (1998 to 2009), the probability of being free of TB was higher in those without diabetes than in those with diabetes (log rank of 392.7281, *p* value < 0.0001) among those who were older than 65 years (Figure 2).

In Table 2, after adjusting for age, sex, income category, residence, gout, hypertension, hyperlipidemia, asthma, COPD, acquired immune deficiency syndrome (AIDS), connective tissue disease, end stage renal disease, heart failure, other cardiovascular disease, anti-hyperglycemic, anti-hypertensive and anti-hyperlipidemia agents, there were lower incidence rates of calcium channel blocker, beta blocker and statin users in the tuberculosis patients aged over 65 years with risk ratios of 0.76 (95% confidence interval (CI), 0.58–0.98 (*p* = 0.0374)), 0.72 (95% CI, 0.58–0.91 (*p* = 0.0048)) and 0.76 (95% CI, 0.60–0.97 (*p* = 0.0291)), respectively. However, the risk of TB was higher in those with advanced age, male sex, residents of southern counties, having asthma, users of acarbose, meglitinides, insulin, diuretics and isosorbide in the tuberculosis patients aged over 65 years with risk ratios of 1.04 (95% CI, 1.02–1.06 (*p* < 0.001)), 1.84 (95% CI, 1.46–2.31 (*p* < 0.0001)), 1.39 (95% CI, 1.03–1.86 (*p* = 0.0288)), 1.83 (95% CI, 1.17–2.86 (*p* = 0.0080)), 1.29 (95% CI, 1.00–1.66 (*p* = 0.0493)), 1.45 (95% CI, 1.14–1.85 (*p* = 0.0026)), 1.42 (95% CI, 1.04–1.96 (*p* = 0.0286)), 1.41 (95% CI, 1.13–1.77 (*p* = 0.0028)) and 1.32 (95% CI, 1.1.02–1.71 (*p* = 0.0323)), respectively.

**Table 2:** Univariate regression and multivariate Cox regression analysis for those >65 years with diabetes with tuberculosis.

Variable	Univariate			Multivariate		
	RR	95% CI	*p* Value	ARR	95% CI	*p* Value
Gout	0.92	0.49–1.73	0.8192	0.93	0.48–1.77	0.8192
Hypertension	0.90	0.68–1.18	0.4281	0.90	0.66–1.22	0.4837
Hyperlipidemia	0.47	0.25–0.88	0.0179	0.57	0.30–1.09	0.0867
Age (mean ± SD)	1.04	1.03–1.06	<0.0001	1.04	1.02–1.06	<0.0001
Sex (Male)	2.02	1.64–2.49	<0.0001	1.84	1.46–2.31	<0.0001
Income category monthly income >NT $20,000	1.40	0.89–2.20	0.1429	1.33	0.84–2.09	0.2206
Northern cities	1.05	0.67–1.64	0.8336	1.01	0.64–1.57	0.9828
Southern cities	0.96	0.54–1.74	0.9027	0.89	0.50–1.61	0.7038
Northern counties	0.94	0.71–1.24	0.6676	0.94	0.71–1.24	0.6480
Southern counties	1.46	1.09–1.94	0.011	1.39	1.03–1.86	0.0288
Asthma	2.07	1.39–3.09	0.0004	1.83	1.17–2.86	0.0080
COPD	1.63	1.18–2.26	0.0031	1.34	0.92–1.95	0.1260
AIDS	-	-	-	-	-	-
Connective tissue disease	0.52	0.13–2.08	0.3533	0.53	0.13–2.15	0.3732
End stage renal disease	0.62	0.16–2.50	0.5042	0.63	0.15–2.50	0.5150
Heart failure	0.98	0.54–1.78	0.9441	0.72	0.38–1.35	0.3035
Other cardiovascular disease	1.31	0.91–1.91	0.1538	1.33	0.89–1.99	0.1652
Sulfonylureas	1.17	0.90–1.53	0.2460	1.30	0.99–1.78	0.0624
Metformin	0.93	0.76–1.16	0.5303	0.93	0.73–1.17	0.5209
Acarbose	1.31	1.04–1.66	0.0215	1.29	1.00–1.66	0.0493
Thiazolidinediones	0.91	0.65–1.27	0.5740	0.88	0.62–1.25	0.4842
Meglitinides	1.50	1.19–1.88	0.0005	1.45	1.14–1.85	0.0026
Insulin	1.50	1.05–1.93	0.0246	1.42	1.04–1.96	0.0286
ACEI	1.08	0.88–1.33	0.4463	1.14	0.92–1.43	0.2380
ARB	0.80	0.66–0.99	0.0362	0.85	0.68–1.07	0.1748
Beta-blockers	0.70	0.57–0.86	0.0008	0.72	0.58–0.91	0.0048
Calcium channel blockers	0.76	0.60–0.96	0.0199	0.76	0.58–0.98	0.0374
Diuretics	1.35	1.09–1.66	0.0053	1.41	1.13–1.77	0.0028
Hydralazine plus nitrate	1.09	0.27–4.37	0.9013	1.23	0.31–4.97	0.7703
All nitrates	-	-	-	-	-	-
Isosorbide	1.24	0.98–1.59	0.0791	1.32	1.02–1.71	0.0323
Other anti-hypertensives	1.42	1.16–1.74	0.0008	1.09	0.87–1.37	0.4617
Statins	0.66	0.53–0.83	0.0004	0.76	0.60–0.97	0.0291
Other anti-hyperlipidemia agents	0.77	0.58–1.03	0.0775	0.93	0.69–1.25	0.6123

ARR = absolute risk reduction; ACEI = angiotensin-converting-enzyme inhibitor; AIDS = acquired immune deficiency syndrome; ARB = angiotensin II receptor blocker; CI = confidence interval; COPD = Chronic obstructive pulmonary disease; RR = risk reduction.

## 3. Discussion

The most important finding in this study is that the users of calcium channel blockers, beta blockers and statins had a lower risk of TB infection in our elderly Taiwanese diabetic cohort. We observed an increased risk of developing TB infection in older males with a history of asthma, users of diuretics, isosorbide, acarbose and insulin-based therapies such as meglitinides and insulin.

It is well known that diabetes mellitus impairs the immunity of patients and therefore is an independent risk factor for TB infection. TB is also a specific morbidity often associated with diabetes mellitus, and is therefore aptly described as a complication of diabetes mellitus [10]. Patients with diabetes are more susceptible to infections and suffer from relatively severe illness due to their immunocompromised status [11], with a higher incidence of reactivation of older foci of TB rather than through fresh contact [12], and they exhibit lower lobe involvement more commonly than patients without diabetes. It has been reported that 5% to 30% of patients with TB also have diabetes [13]. One review [14] of nine studies found that diabetes was estimated to increase the risk of TB by 1.5- to 7.8-fold, and a meta-analysis demonstrated that diabetes was associated with a relative risk of 3.11 for contracting TB [15]. Furthermore, a study from America observed that multi-drug resistant TB was associated with diabetes mellitus with an odds ratio of 2.1 [16].

An association between hypertension and activation of the immune system has long been recognized. Studies of human hypertension support this association and implicate a mechanistic role of immune activation and inflammation in the development of hypertension [17,18,19,20,21]. Experimental animal models have proven especially useful for determining the impact of specific immune cells (innate and adaptive immunity) and cytokines, with a heavy emphasis on angiotensin II (AngII)-dependent hypertension in rodent models [22,23,24,25,26]. As blood pressure is regulated by a combination of the kidneys, central nervous system, and vasculature, the impact of the immune system on the renal and central nervous systems as well as on vascular function is strongly associated with an imbalance between pro- and anti-inflammatory pathways that lead to the accumulation of immune cells (*i.e.*, T cells and macrophages) in tissues [27]. The presence of these cells can increase the local production of inflammatory cytokines and activate signaling pathways such as NF-κB, leading to oxidative stress that further perpetuates declining organ function [28]. It has recently been reported that during TB infection, an increase in calcium influx or release of calcium from intracellular calcium pools activates the intracellular calcium-signaling pathway, thereby activating the gene expressions of anti-infection and immune-protective proteins in immune cells, and especially macrophages [29,30]. This increase in calcium signaling enhances the phagocytic activity and anti-TB ability of immune cells, ultimately enhancing the anti-TB ability of the whole immune system. As the key player in maintaining intracellular calcium levels, calcium channels play a crucial role in regulating the calcium-signaling pathway. Calcium channel blockers enhance the intracellular calcium level and trigger the downstream calcium-signaling pathway, ultimately activating anti-infection gene expressions [29]. In the current study, calcium channel blockers were shown to decrease the risk of developing TB infection in our elderly patients.

Most immune cells have beta-adrenoreceptors that are used by catecholamines to regulate their functions. Given that norepinephrine and epinephrine modulate immune function and that many of these effects are blocked by beta-blockers, the inhibition of their receptors will affect the immune response and the cells involved therein [31]. A common dogma of the 1980s and early 1990s was that norepinephrine stimulation suppressed lymphocyte function. However, alterations in the expression of lymphocyte adhesion molecules may mediate the norepinephrine-induced increase in the number of circulating lymphocytes [32]. Therefore, the intake of beta blockers can also alter the flux of immune cells. Beta blockers interfere with the adrenergic regulation of the number of circulating leukocytes by blunting psychological stress effects while enhancing the effects of exercise [33]. Noradrenaline and adrenaline at high concentrations stimulate T lymphocyte production, however this stimulation is inhibited by beta blockers. On the other hand, high concentrations of noradrenaline inhibit the proliferation of lymphocytes, which regulate the proliferative response [34]. Beta blocker treatment in the healthy can alter the immune equilibrium, especially during stress situations such as exercise. In the current study, beta blockers were shown to decrease the risk of developing TB infection in elderly Taiwanese patients.

Statins may prevent TB by reducing cholesterol. *In vitro* studies have demonstrated that statins (HMG-CoA reductase inhibitors) reduce macrophage cholesterol by multiple mechanisms, including reducing cholesterol biosynthesis, stimulating cholesterol efflux and inhibiting cholesterol ester accumulation [35,36]. *In vitro* studies have also reported that statin treatment reduces phagocytosis in macrophages due to a cholesterol lowering effect on macrophages [37]. Therefore, there is enough evidence to suggest that the cholesterol lowering property of statins contributes to the prevention of TB, since host cholesterol is an important biomolecule for successful TB infection. A low concentration of serum vitamin D3 has been reported in patients suffering from active tuberculosis [38], implying that vitamin D has an influence on the immune response to TB. In addition, vitamin D deficiency has been associated with an increased risk of tuberculosis in different populations [39]. Since statins are known to inhibit cholesterol production, they would be expected to reduce vitamin D3 concentration since it is a downstream product of cholesterol. Macrophage membrane cholesterol is found more often in patients with diabetes, and may thus also be an important cause of an increased occurrence of tuberculosis in patients with diabetes [40]. Taken together, statin therapy can effectively reduce macrophage cholesterol and thereby reduce the risk of TB in patients with diabetes. Moreover, vitamin D has recently been reported to improve the diabetic state and positively influence pancreatic beta-cell function [41]. Therefore, statin therapy for patients with diabetes may improve insulin secretion by increasing vitamin D synthesis, which in turn can improve the diabetic state resulting in a reduced risk of TB. Vitamin D synthesized by statins is expected to reduce vitamin D deficiency in patients with diabetes [42], and that should play a beneficial role in the context of tuberculosis. In the current study, statins were demonstrated to decrease the risk of developing TB infection in the elderly.

Patients diagnosed with TB may have extensive co-morbidities including asthma. Asthma is a common chronic inflammatory disease of the airways characterized by variable and recurring symptoms, reversible airflow obstruction and bronchospasm. Airflow limitation is usually both progressive and associated with an abnormal inflammatory response of the lungs to noxious particles or gases [43]. Susceptibility to the development of active tuberculosis and asthma involves a complex interaction between genetic and environmental factors, which is at present poorly understood. The chronic inflammation of the airways subsequently results in increased contractability of the surrounding smooth muscles, an increase in eosinophils and thickening of the lamina reticularis. In chronic asthma, the smooth muscles of the airway may increase in size along with an increase in the number of mucous glands. Other cell types involved include T lymphocytes, macrophages, and neutrophils. Other components of the immune system may also be involved, including cytokines, chemokines, histamine, and leukotrienes among others [44]. Glucocorticoids used in the treatment of asthma should be considered a risk factor for developing TB, as systemic glucocorticoids have profound effects on the cellular immune response which controls TB. Glucocorticoids inhibit the lymphokine effect and monocyte chemotaxis and also block Fc receptor binding and function [45]. They lower the number of peripheral blood monocytes as well as monocyte function including bactericidal activity and the production of interleukin-1 and TNF-α [46], and also inhibit T cell activation, leading to reduced proliferative response and cytokine production. Furthermore, they also induce a redistribution of lymphocytes (predominantly T cells) out of the circulation, leading to peripheral lymphocytopenia [47]. In the current study, the presence of asthma led to an increased risk of developing TB infection.

Atherosclerosis, the major cause of cardiovascular disease, is a chronic inflammatory condition with immune competent cells in lesions producing mainly pro-inflammatory cytokines, and it occurs mostly in elderly patient as part of the aging process. Bacteria and viruses have been proposed to cause atherosclerosis, however there is little direct evidence and antibiotic trials have not been successful. Infections have also been proposed to be a potential cause of immune activation and inflammation in atherosclerosis [48]. Isosorbide, a long-acting metabolite of the vasodilator isosorbide dinitrate used for the prophylactic treatment of angina pectoris, is known to have adverse reactions including bronchitis, pneumonia and upper respiratory tract infections [49]. In the current study, the use of isosorbide increased the risk of developing TB infection.

Some diuretics such as furosemide and spironolactone are potent inhibitors of leukocyte migration through endothelial cell monolayers [50]. The anti-inflammatory effects of spironolactone on human peripheral blood mononuclear cells have also been described [51]. These effects may compromise the host defense during an infection, and especially during exercise and stress. In the current study, the use of diuretics increased the risk of developing TB infection.

Trehalose is a non-reducing disaccharide of glucose which is used by many lower organisms such as mycobacteria for key functions such as energy storage, signaling, protein-protection and bacterial cell wall components [52]. In mycobacteria, trehalose is also part of a toxic lipid in the cell wall known as trehalose-6,6'-dimycolate or cord factor, which has been identified as the main virulence factor of tuberculosis [53]. Accordingly, considerable attention has been directed toward the possibility that enzymes involved in the production of trehalose may serve as drug targets. Trehalose synthase catalyzes the reversible conversion of maltose into trehalose in mycobacteria to this non-reducing disaccharide. Previous studies have suggested that trehalose synthase exhibits α-amylase activity, which can be competitively inhibited by the potent α-glucosidase inhibitor, acarbose [54]. In other studies, however, acarbose has been reported to bind to a remote site that is unlikely to possess amylase activity given the lack of appropriate catalytic residues in its binding pocket [55]. This casts some doubt on the proposed inherent amylase activity. In the current study, the use of acarbose increased the risk of developing TB infection.

The management of diabetes mellitus in patients with TB should be aggressive. Insulin therapy should be initiated at the outset, using a basal bolus regimen or premixed insulin [56]. The American Association of Clinical Endocrinologists recommends the use of modern insulin or insulin analogues, whereas the use of traditional human insulin is discouraged [57]. A large, population-based, cohort study including 19.9 million residents of Australia with adjustments for important confounding factors reported that people with diabetes mellitus had a 1.5-fold increased risk of developing TB, however the number of cases of TB attributable to diabetes mellitus was very small; in addition, those using insulin for diabetes mellitus had a greater risk of TB [58]. In the current study, insulin-based therapies including meglitinides and insulin increased the risk of developing TB infection.

There are several methodological strengths and limitations to this study. First, this study was prospective in design, had a long follow-up period, and a very large cohort of patients with diabetes and tuberculosis. Second, the diagnoses of TB and diabetes were likely to be accurate as they were obtained from computerized data files for each individual from the NHIRD, which is population-based and highly representative, causing little possibility of recall and selection bias. Nevertheless, there are some limitations. First, medication exposure was based on prescription information only, and thus we could not determine whether the study patients actually adhered to the prescribed dosage schedule. This bias may have caused random misclassification of exposure and underestimation of our findings. In addition, medication can be purchased easily over the counter, although, in Taiwan, such purchases are reduced by the NHI system that covers the cost of almost all drugs and allows patients to visit almost any physician that they choose. This bias may have affected the analysis, however the effect was probably minimal. Second, no laboratory data are recorded in the NHIRD, and the diagnosis of diabetes was not based on clinical criteria. In addition, we did not include several important clinical confounders such as body mass index, HbA1C, duration of diabetes, smoking, alcohol consumption in the models to assess the incidence of diabetes. Instead, we used a conservative method to define diabetes, but one by which the accuracy of identifying cases reached 96.1%. Finally, unmeasured covariates may have correlated with the exposure of interest and affected the outcome of interest in this study.

## 4. Materials and Methods

### 4.1. Setting

This study was funded by the Department of Internal Medicine, Kaohsiung Medical University Hospital to use data from the National Health Insurance Research Database (NHIRD), published by the National Health Research Institute (NHRI) in Taiwan, which includes data for one million randomly selected subjects who were followed from 1998 to 2009. The NHI program was implemented in Taiwan in 1995 and offers a comprehensive, unified, and universal health insurance program to all citizens, including those who have established a registered domicile for at least four months in the Taiwan area. The coverage provides outpatient services, inpatient care, Chinese medicine, dental care, childbirth, physical therapy, preventive health care, home care, and rehabilitation for chronic mental illnesses. The coverage rate was 96.16% of the whole the population in 2000, rising to 99% at the end of 2004. The NHI medical claims database includes data on ambulatory care, hospital inpatient care, dental services, and prescription drugs. The state-run Bureau of National Health Insurance (BNHI) has contracts with 80% to 97% of hospitals as well as 90% of the clinics across Taiwan. The BNHI accumulates all administrative and claims data for Taiwan, and the NHRI cooperates with the BNHI to create the NHIRD, which consists of cumulative information on one million randomly selected subjects including personal identification number (PIN), date of birth, sex, geographic area of the NHI unit, and dates of enrollment and withdrawal from March 1995 to December 2009 [59]. There are no statistically significant differences in age, sex, and average insured payroll-related amount in all enrollees. With regards to the accuracy of the claims data, the BNHI performs quarterly expert reviews on a random sample of every 50 to 100 ambulatory and inpatient claims in each hospital and clinic. Given that the claim data includes both inpatients and ambulatory patients, we believe that this recruitment procedure would avoid bias with regards the health status of the patients. Although it was not easy to assess the sensitivity and specificity of this approach, including the disease duration, both were believed to be good because the clinical setting meant that all cases of diabetes in the claims data tended to be reported to ensure full reimbursement, and because reporting a false diagnosis would incur a severe penalty from the BNHI. With ethical approval from the NHRI, we used data from the ambulatory care claims, all inpatient claims, and an updated registry of beneficiaries from 1998 to 2009 for this study. All of the data sets could be linked via individual PINs, however the identity of the individual was protected via encryption. Since the diagnostic coding of the NHI in Taiwan follows the International Classification of Diseases, Ninth Revision, Clinical Modification (ICD-9-CM) diagnostic criteria, patients with diabetes were identified by an ICD-9-CM code of 250 (excluding type 1 diabetes mellitus, which is ICD-9-CM code 2501). An elderly diabetic patient was defined as a patient aged 65 years or older with an initial diabetes-related diagnosis at any time in 1998, and then with at least one service claim for either ambulatory or inpatient care within the subsequent 12 months. The diagnosis of diabetes was further confirmed by treatment at baseline including two or more monthly prescriptions of hypoglycemic medications prescribed by validated physicians. To identify newly diagnosed cases of TB, we excluded patients diagnosed with any type of TB (ICD-9-CM codes 011–018) during 1995–1997 from our study group. We defined active TB using compatible ICD-9-CM codes of TB plus the prescription of more than two anti-tuberculosis medications for more than 28 days. The index date for patients in the diabetic group was the date of the first outpatient visit for diabetes care in 1998.

### 4.2. Study end Points

The ambulatory and inpatient claims included records of all hospitalizations and information on PIN, date of birth, sex, dates of admission and discharge, a maximum of five leading diagnoses and four operation codes, and the proportion of the cost paid by the beneficiary for the admission. With the unique PIN, we linked study subjects in both the diabetes and non-diabetes groups to ambulatory and inpatient claims data from 1998 to 2009 to identify, if any, the first diagnosis of any type of TB infection (ICD-9-CM 011–018) as the end point of this study. In Taiwan, TB has been a mandatory modifiable disease for a number of decades, and physicians are required by law to report patients with TB and whether they are incident or recurrent cases. The Taiwan Center for Disease Control maintains a web-based electronic database as a national TB registry, which includes the patient’s unique identification number and relevant bacteriological and clinical information. In Taiwan, all health care services for TB are reimbursed through the NHI. To improve the notification of TB, the BNHI introduced a policy of “no notification, no reimbursement” in 1997 for related cases of diabetes, hypertension, dyslipidemia, and gout. A recent analysis using a cross-matched database of TB notification and NHI reimbursement found that over 96% of TB patients were registered in the TB registry [60]. Following national TB guidelines, a case of pulmonary TB was defined by a positive sputum smear or sputum culture or, in the absence of bacteriological evidence, positive chest X-ray compatible with pulmonary TB plus clinical improvement after anti-TB treatment [61]. This study was conducted from 1 January 1998 to 31 December 2009, an 11-year-period.

### 4.3. Possible Associated Risk Factors of Diabetes and Tuberculosis Infection

We identified possible associated risk factors of diabetes and tuberculosis infection including hypertension (ICD-9-CM: 401 and 405), dyslipidemia (ICD-9-CM: 272), gout (ICD-9-CM: 274), asthma (ICD-9-CM: 493), COPD (ICD-9-CM: 490–492, 496 and 5064), AIDS (ICD-9-CM: 042–044), connective tissue disease (ICD-9-CM: 710, 711 and 714), end stage renal disease (ICD-9-CM: 585), heart failure (ICD-9-CM: 428) and other cardiovascular diseases (ICD-9-CM: 430–438) from ambulatory and inpatient claims data (1998–2009). These possibly associated risk factors were included in the analysis only when the date of diagnosis was before or on the day on which the study subjects were diagnosed with diabetes, and considered to be baseline disease or baseline health status of the studied population. The study subjects who had at least one service claim for either ambulatory or inpatient care within the subsequent 12 months with a primary diagnosis of hypertension, dyslipidemia, or gout without any type of tuberculosis infection were identified in 1998. The diagnoses of hypertension, dyslipidemia, and gout were further confirmed by treatment at baseline by validated physicians who prescribed two or more monthly prescriptions of anti-hypertensive, anti-hyperlipidemia, and anti-gout or anti-hyperuricemic agents in 1998.

### 4.4. Statistical Methods

We performed two major statistical analyses in this study. First, differences in demographic characteristics between the patients with and without diabetes, including age, sex, income category, residence, hypertension, dyslipidemia, gout, asthma, COPD, AIDS, connective tissue disease, end stage renal disease, heart failure, other CV disease and tuberculosis were analyzed using the χ2 test. Second, to determine the independent effects of diabetes on the risk of tuberculosis infection, we used Cox proportional hazard regression models with age, sex, and the possibly associated risk factors adjusted for simultaneously in the model. Furthermore, we explored the relative hazards of tuberculosis infection in relation to diabetes accompanied by the selected possibly associated risk factors which were already present before the diagnosis of diabetes individually using Cox proportional hazard regression models with adjustments for all types of anti-hypertensive, anti-hyperlipidemia and anti-hyperglycemic agents in the model. All statistical analyses were performed with SAS version 9.1 (SAS Institute, Cary, NC, USA). A *p* value <0.05 was considered to be statistically significant.

## 5. Conclusions

Due to the aging population and increasing incidence of diabetes, both diabetic patients and also patients with asthma should be strongly encouraged by physicians to undergo appropriate TB screening. Because most epidemiologic studies have focused on confounding factors such as the metabolic syndrome and hypertension and dyslipidemia, which commonly coexist with diabetes, the choice of treatment should warrant public health attention.
